# A family systems approach to genetic counseling: Development of narrative interventions

**DOI:** 10.1002/jgc4.1377

**Published:** 2021-01-12

**Authors:** Rhona MacLeod, Alison Metcalfe, Mariangels Ferrer‐Duch

**Affiliations:** ^1^ Manchester Centre for Genomic Medicine St Mary's Hospital Manchester University NHS Foundation Trust Manchester Academic Health Sciences Centre Manchester UK; ^2^ Division of Evolution and Genomic Sciences School of Biological Sciences University of Manchester Manchester UK; ^3^ Faculty of Health and Well‐being Sheffield Hallam University Sheffield UK; ^4^ Riverbank Psychology Manchester UK

**Keywords:** Counseling techniques, family, genetic counseling, multi‐disciplinary

## Abstract

To what extent are family systems approaches relevant in the genomics era? What difference does it make to remember the wider social context within which ‘problems’ associated with a genetic diagnosis reside? How does this influence the conversations we have with our patients? These questions will be considered in relation to systemic approaches to genetic counseling practice. Narrative therapy with an emphasis on people's strengths, wishes, and ways of resisting the effects of a problem may be a particularly useful framework for genetic counselors. Narrative practice views people as multi‐storied and is concerned with the question of how we encourage people to tell their stories in ways that make them feel stronger. Increased uptake of genomic testing and the number of people seeking genetic counseling present opportunities to consider new ways of working, particularly around support following a new genetic diagnosis. One option is to realize the potential of group interventions. Family therapy and narrative practices have the potential to encourage communication and for families to learn from each other.

## 1 INTRODUCTION

Advances in the science of genomics and its relevance for the wider community suggests a utility in looking at systemic ways of working and what it means to be at risk of a health‐threatening condition. How can we translate this new understanding? How might it shift the conversations around rare genetic conditions in such a way as to help us all feel more connected? The co‐authors in this paper (MF‐D and AM), drawing on their expertise in the disciplines of clinical and health psychology and nursing, have influenced our perspectives on how we might adapt systemic ways of working to genetic counseling practice. In this paper, we will consider why family systems approaches may be useful for genetic counselors in their work. We will focus on a particular model, narrative therapy, as one that has underpinned counseling supervision and training for genetic counselors in Manchester Centre for Genomic Medicine since 2012.

## 2 WHY FAMILY SYSTEMS APPROACHES?

Family systems thinking views the family as an emotional unit, and it uses a systems approach to describe their complex interactions. Family members are strongly interconnected and a change in one person's experiences or emotions is followed by reciprocal changes in others (Kerr, 2000). Therefore, diagnosis of a genetic condition or a test result is multi‐layered in its reverberation within the family because the patient (or proband)'s experience or emotions will have an impact for other family members, but there might also be consequences of the genetic testing for other family members.

Systems approaches are not new to genetic counseling; the ‘patient’ is seldom an individual, and even when seen alone, genetic counselors are mindful of the genetic and psychosocial impact of a genetic condition on the wider family visualized in every consultation through the family tree (Gaff et al., 2007; Mendes et al., 2016). Seymour Kessler (1979) advocated the need for genetic counselors (GCs) to understand patients’ problems in context, taking account of their life histories (including values and goals), and as members of social groups both in relation to their own family and as members of a wider social group (Kessler, 1979).

Communication of genetic risk information within families is, however, often challenging (Forrest Keenan et al., 2013; Metcalfe et al., 2011). While complete non‐disclosure is uncommon, partial disclosure or reluctance to revisit initial conversations about a genetic condition appears to be more common (Goldman et al., 2018). Systemic approaches are important here in understanding a ‘problem’ in context and as relational rather than clinicians seeing it as a ‘failure’ on the part of the proband, to disclose.

Further need for systemic approaches comes from advances in genomic technology and increase in genomic testing with blood samples often required from other family members in order to determine the likely pathogenicity of a particular genetic variant (Ormond, 2013; Tsai et al., 2020).

Family systems theory is a commonly cited model in the genetic counseling literature. This extends from single cases studies (Sobel, 2005; Werner‐Lin Gardener, 2009) and reviews of the literature (Mendes et al., 2016) to complex interventions such as Multi‐Family Discussion Groups (Eisler et al., 2017; Mendes et al., 2010; SPRinG Collaboration, 2016).

In earlier work, Metcalfe et al. (2008), Metcalfe et al. (2011) identified that many families often felt quite isolated by their genetic diagnosis, were struggling to communicate about the condition in the family, and reported wanting more support, which was not forthcoming from health services in the UK. Many of the families interviewed including parents and children described the strain of living with the genetic condition or its risk—with the potential for families to experience more serious problems. After consulting with families and professionals, an intervention was developed based on Multi‐Family Discussion Groups (MFDG), which puts emphasis on families learning from each other and also recognizing their own strengths and capabilities in managing complex emotions and situations (SPRinG Collaboration, 2016).

The authors have a collective interest in interventions that help to move people from a position of isolation or potential isolation. This paper will now focus on one particular model, the Narrative model, and consider its potential adaptation for genetic counseling.

## 3 THE NARRATIVE MODEL

The Narrative model can trace its origins to the ideas of Michael White and David Epston developed over many years of working with families in the context of family therapy and social work (White & Epston, 1990). Theoretical influences of the narrative model include anthropology, feminism, philosophy, psychology, community work and activism, and social justice (Freedman & Combs, 1996; Madigan, 2010; Russel & Carey, 2004; White, 1997).

## 4 PRINCIPLES OF NARRATIVE PRACTICES AND RELEVANCE FOR GENETIC COUNSELING

Narrative practice is a collaborative way of working that recognizes that people's lives are made up of multiple stories which can be richly described. With the narrative resources, the genetic counselor can contribute to generate new meanings that the person might give to their experience of the genetic condition. From this perspective, it is understood that people are experts in their own lives and have many skills, experiences, hopes, and values to draw on (White, 2007; White & Epston, 1990) that can help them adapt to living with a genetic condition or its risks. As such, narrative expertise is not about delivering ‘interventions’, but instead providing the possibility of multiple contexts, through scaffolding of questions that allows people to become more aware of their own preferences, skills, and knowledge, and indeed recognize, sometimes long‐forgotten personal and family stories that they can draw on to see themselves in a new or different light. Once patients are in touch with their own skills, they are then able to use these in order to address difficulties they may be facing.

## 5 EXTERNALIZING CONVERSATIONS

A central idea in working within the narrative model is ‘externalizing conversations’. Externalizing can be considered a linguistic practice and locates problems as relational and separated from people. This way of seeing the problem, objectifies the problem and not the person. As a summary, we can say that ‘the person is not the problem, the problem is the problem’. This way of seeing problems involves taking a non‐judgmental position toward people.

In the context of genetic counseling, we find that ‘the problem’ is not only ‘the problem’ but on many occasions ‘the effects of the problem’. For example, a person with myotonic dystrophy may be concerned about the uncertainty of the condition or the tiredness associated with it. When externalizing is at the center of conversations with a genetic counselor, patients are provided with the space to reconsider their position in relation to what seems problematic in their lives. Using the same example, a narrative therapist would explore with the person if they can give a name to their ‘problem’. In this case, the person may identify this as ‘tiredness’. Questions can then be asked about the effects of the ‘tiredness’ in his or her life. We can ask questions like, where is ‘tiredness’ more present in your everyday life? Is ‘tiredness’ more present in the morning or in the evening? Is ‘tiredness’ getting in the way of something important? Are you able to stop the effects of ‘tiredness’ at work sometimes? Is there a person that helps you to diminish the effects of ‘tiredness’? In this way, it becomes more possible to identify skills that the patient has and can develop to resist the effects of the problem. Furthermore, it becomes possible to find other important people that can form a team of support and sustain them in the process of reducing the negative influence of the problem (Russel & Carey, 2004; White, 2007).

## 6 DOUBLE LISTENING

Another influential idea from Narrative therapy in working in the genetic counseling context is the position that narrative practitioners take on trauma and traumatic events.

No‐one is a passive recipient of hardship. People are always responding, whether they are children or adults. They respond to try to minimize the effects of hardship, or to try to make it stop, or to try to protect others, and so on. These responses are often overlooked or disqualified – so much so that people are often not familiar with their own responses. (Denborough, 2010)

The listening for the problematic story and the responses to the problematic story is called the practice of double listening (White, 2003). We listen to both the story of the problem and also any small acts that have resisted and helped to move away from the effects of the problem.

Double listening provides a helpful way of thinking about families for whom receiving a genetic diagnosis has been a difficult experience or where living with a genetic condition is experienced as ongoing trauma, we can invite individuals and families to observe the way they have been responding or protecting themselves from the effects of the condition. On too many occasions, when facing problematic or overwhelming circumstances as human beings we stop noticing the way we respond or recognizing that we have ways to keep going. Part of the role of the genetic counselor can be to notice the small actions that are taking place in the lives of families that are parallel to the hardships that they are experiencing in relation to the genetic condition. This exercise positions patients as active actors in their lives and not victims or at best survivors of a genetic condition (Fox, 2017). Narrative practices encourage us to be curious about these actions, however subtle or small; they are there to be discovered and require a particular way of listening. For example, once we hear that an action has happened as a response to a problematic situation or regardless of the problematic situation, we can ask questions like ‘why was this important to you?’ ‘what do you think made this possible?’ and ‘what difference do you think it made to your son that you were still able to pick him up from school?’ When the focus is on the small actions that people are taking away from the problem, it can help to energize the session. Typically, the patient may tell you that they have not thought about it before or appear surprised themselves by their own response. This can be the beginning of a different type of conversation, a conversation where the patient feels empowered by his or her own resources and strengths. We are influencing the patient's new understanding of things while not assuming to know what is best for them.

Listening for ways people resist the effects of a problem is not new and will be familiar to genetic counselors. We may for example reflect back the words a patient has used or acknowledge what we have heard for example in a consultation with a woman with a BrCa‐associated breast cancer, ‘so even when you were feeling unwell from the chemotherapy and “low” you continued to pick up your son from school’. It is about both recognizing the ‘feeling unwell from chemotherapy’ and at the same time making visible and available the apparently small detail that the person, parallel to feeling unwell, has been able to ‘pick up her son from school’. This becomes even more powerful and transformative when it is given back to the patient to evaluate. The GC can then be curious about the action of picking up the child from school—‘Is picking up your son from school something important for you?’, ‘Why is this important?’, and ‘What do you think it says about you that even though you were feeling unwell you decided to pick up your son from school?’ The responses help to create a new narrative that runs parallel to the narrative of ‘feeling unwell from chemotherapy’ and shines a light on the patient's resources, providing a point of entry to conversations around hope and what is still possible to do. From our practice experience, we see in the non‐verbal responses of patients (smiling, relaxed body posture) together with their verbal feedback that this new understanding is bringing possibilities and a new sense of having more control of their lives. We keep checking in the conversation—‘How is this consultation going? and Are we addressing what is important for you to address?’ In this way, each conversation with a patient contributes to the skills’ development (White, 1997, p. 138) of the narrative therapist or genetic counselor.

## 7 EXAMPLES FROM PRACTICE: GENETIC COUNSELING WITH FAMILY, GROUP WORK, AND PUBLIC ENGAGEMENT

### 7.1 Example from genetic counselling practice

#### 7.1.1 Mary Reid and Tom Brown

**FIGURE 1:**
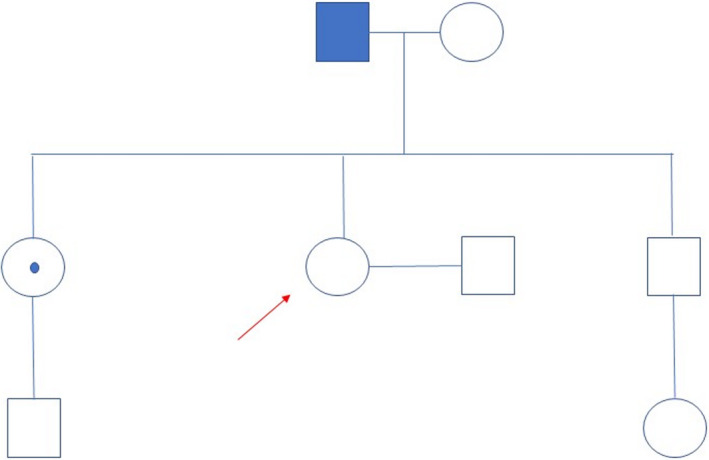
Reid family tree

Mary Reid (names changed) age 38 recently moved to Manchester with her partner Tom and started a new job as a charity organizer. Her dad, James Reid age 68, had been diagnosed the previous year with Huntington's Disease (HD). The family had all attended their local genetics department. Mary and Tom had been together for 4 years and were on the point of trying for a family when the genetic diagnosis in Mary's dad came ‘out the blue’. The family had been aware that James was unsteady on his feet and becoming increasingly irritable but there was no known family history of similar problems (Figure 1).

Mary's older sister Lesley, who lives in another city, had been keen to pursue predictive testing soon after finding out about HD and was given the result 2 months ago that she carries the HD gene fault. Mary had been feeling guilty about moving away and leaving her mum to cope with looking after her dad, was anxious about her sister's test result, and feeling very uncertain about what to do in relation to plans for starting a family. She had been thinking about having a predictive test as a way of clarifying her thoughts about reproductive choices but was unsure that she felt ready to deal with a test result. Mary and Tom attended the clinic together and came with letters and printed information from the genetics clinic they had attended prior to the move.

In addition to understanding the background to how the referral came about and how Mary and the family were coping with the new diagnosis, I was interested in what Mary was telling me in relation to the recent move. Mary explained that Tom had secured a promotion and that this had been the prompt for the decision to relocate. Neither of them knew anyone in Manchester at the time but had a good network of friends around the country. Mary mentioned that she had also started a new job in a nearby hospital. As a genetic counselor, we frequently hear amazing small (and not so small!) accomplishments while people are facing challenges in their lives. A counseling approach would be to acknowledge and validate this achievement, perhaps applauding the achievement or reflecting what had been heard, for example, ‘so even while all this was going on you were able to secure a new job?’

Adopting a more narrative position, I was curious about how Mary was able to achieve this rather than accept what she was telling me at face value. Most importantly, I was checking that she was happy with what seemed an amazing accomplishment. I asked Mary questions like: What steps made this possible?, What other strengths/things/people were involved in supporting this course of action?, and Who else would not have been surprised that you were able to achieve this?

The following is an extract from the conversation at this point in the conversation:

in the midst of all that was happening at home, how did you manage to make the move? What thing (s) helped to make this possible?

The question seemed to surprise Mary, and on reflection may have been too big a question. It did have the effect of releasing the tension and both Mary and Tom laughed in agreement.

I don't know…….lots of red wine!

At the point where I was wondering how I might break the question down more, I noticed that Mary was now quiet and appeared very thoughtful. After a moment or two's silence, she said:

Actually, I do know what is – I'm the sort of person who knows what they want

At this point, Tom agreed and reinforced the idea that it was something he recognized in Mary.

This felt like a ‘pearl’ in the consultation. The mood in the room had changed, and I was wondering how we could use this re‐found knowledge to help them with their dilemma about options for starting a family. Before this could even be posed to them as a question however, Mary said in a clear tone:

I do know what I want – I want to have a baby!

It seems that the questions helped to re‐connect Mary not just to her values and commitment about how important it was for her to have a baby, but also the existing skills/strengths she had to make this happen. The consultation in fact ended quite soon after this exchange and Mary seemed energized leaving the room. Before leaving, Mary disclosed that one of the things that had been making her feel ‘quite bad’ in the last year was that she did not feel as though she had been coping very well.

you know I've actually been feeling quite bad that I don't seem to be coping very well but I have actually been doing things…

The narrative‐style questions were able to help shift how Mary saw herself in relation to the situation she was in and the things that she had been doing in spite of HD in their lives.

This was probably among the most dramatic effect of narrative‐style questions I have witnessed in a consultation. Among my GC colleagues though we have all noticed how the narrative stance and timely narrative questions can bring about a shift in the consultation, often lightening the tone with patients sometimes surprised both by the questions and their own responses.

Mary would likely have reached this same point in different types of accompanied genetic counseling conversations. Nevertheless, it was surprising the speed with which this discovery turned the session around from distress about the diagnosis of HD, to a plan of what she wanted to do next. It is not to say that the distress in relation to HD went away, because adopting a narrative approach is not about ignoring the effects of the problem. It is possible, however, when accompanying patients who are very upset that we may miss these smaller actions or responses to what they perceive to be the problem.

Mary and Tom seemed clear about their next steps and indeed eighteen months on they have a son conceived by egg donation.

## 8 GENETIC COUNSELING NARRATIVE GROUPS

A means to talk, a key to use in a lock, to express my thoughts and feelings (without too much emotion slowing me down). (Participant in a genetic counseling narrative group)

Since 2015 as part of a service improvement, we have been offering Genetic Counseling narrative groups as a stand‐alone session to small groups of patients (MacLeod et al., 2018; Spiers et al., 2020; Stopford et al., 2020).

This came about through the experienced benefits of receiving narrative counseling supervision as well as experiencing the effects on our patients of integrating narrative ideas in to our GC sessions. In common with most genetic counselors, we have a high demand on our follow‐up appointment slots in clinic. It raised the question of whether a group session could be useful in particular situations for example post‐predictive test follow‐up.

These group sessions were initially offered to patients with a family history of Huntington's disease and subsequently evaluated with other groups of patients including groups for familial cancer, cardiac, and eye genetic conditions (MacLeod et al., 2018; Spiers et al., 2020; Stopford et al., 2020). The aim of these groups is to help support resilience and participants’ own preferred ways of coping.

**FIGURE 2:**
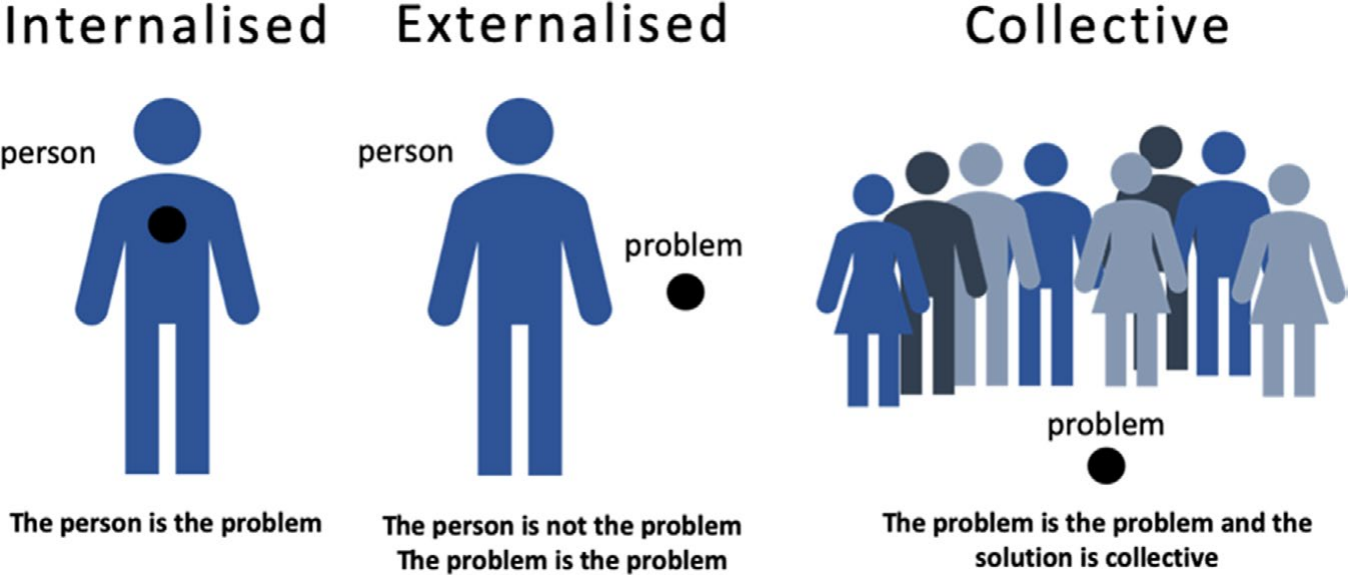
Externalizing conversations collective

The group sessions embrace the premise from Narrative therapy that if the problem is not the person, the solution therefore must be collective (Denborough, 2008; Figure 2).

These groups have evolved over time, driven largely by the feedback from participants who welcomed both the chance to meet with others with a similar situation in the hospital setting and to combine a genetic counseling appointment with an exercise to make more visible their own strengths. Between October 2015 and May 2019, we conducted 15 groups detailed in Table 1 below.

**TABLE 1:** Genetic counseling narrative groups

Clinical teams	Narrative groups	No of participants
Neurogenetic	9	53
Eye	2	10
Cardiac	1	6
Cancer	1	4
Developmental disorders	2	10
Total	15	83

Each narrative group is co‐facilitated by a psychologist (M F‐D) and a genetic counselor, usually known to the participants already. Participants only attend a narrative group once. The session lasts two hours and is structured with a short break. The structured narrative exercise is an adaptation of the Tree of Life exercise (Ncube, 2015). Essentially, the structure of the tree's metaphor is used to layer a series of narrative‐style questions to elicit people's skills, strengths, hopes, and existing relationships. It is also our intention to facilitate space for the group where new knowledge and abilities in relation to living with a genetic condition will emerge. We never know before we start how each group will discover and share these new understandings. We assume that families and individuals have a good amount of resources and knowledge about their lives (White, 2003) and have a rich understanding and lived experience of their family's condition. Patients have found the groups still relevant when they have had news of their genetic condition relatively recently. We provide the context based on the shared condition or type of genetic conditions of patients and the experience of the GC's individual conversations with them.

Spiers et al. (2020) conducted a qualitative study using Interpretative Phenomenological Analysis (IPA) to interview 12 people (10 women and 2 men) who had taken part in a Genetic Counseling Narrative Group between November 2017 and February 2018. The age range was 27–67 with median age of 36 years. Telephone interviews were conducted with participants 2–4 weeks following their attendance at a group session. Of the 12 people interviewed, most were very positive about their experiences of participating, not just in terms of what they experienced themselves but ways they felt they had contributed and bolstered other people in the group. Two participants were less able to identify benefits for themselves in taking part. For the 10 participants who felt the group had had an impact following the session, this related to feeling more able to talk about the genetic condition with friends and family and noticing a positive effect on their thoughts and feelings.

## 9 PUBLIC ENGAGEMENT

### 9.1 Narrative practices: Extending the conversations about rare genetic conditions

In a more recent departure, extending a narrative approach to conversations about rare genetic conditions, we have teamed up as a small multi‐disciplinary group to record a series of podcasts. https://rare‐d.com

We hope that by including narrative‐style questions in our interviews with guests on subjects such as the 100 k genome project and antenatal screening, we can encourage each of us to consider our own connection to rare conditions. A final question at the end of each podcast invites guests to think about, ‘What does “rare” mean to you?’ We hope to evoke multiple answers and images to this question with the intention to bring to the conversation the not so mainstream understandings of what rare and rare disease means for many people.

This project has also given us an opportunity to connect with colleagues and patients in countries such as India, Romania, Italy and China. In those conversations, we adapted an outsider witness approach (Myerhoff, 1986; White, 2003). This approach emphasizes the practice of double listening (mentioned before), highlights the connection between people, and facilitates linking lives to common themes. In this way, the guest at the center would be interviewed by the hosts (Mariangels Ferrer‐Duch and Nichola Garde) and two or three additional guests become the witnesses guests that would listen to the conversation and be invited to comment on what they had heard—a word or phrase that caught their attention that might evoke an image and how this resonated with them. They were also invited to consider what they would take away from the conversation/what they might do differently as a consequence of having heard and being part of the conversation. In this way, many of the alternative themes of the conversation are further developed, and the shared stories become linked through these themes, and through the values and commitments expressed in them. This structure is validating our guest's preferred claims about their positions, and they hopefully contribute to options for action in both our guests and our audience (White, 1997).

The outsider witness (OW) approach may be adopted in narrative counseling supervision (Ferrer‐Duch, 2017). Indeed, the structure of the recent podcasts mirrors the OW approach of our monthly GC group counseling supervision. One of the genetic counselors in the group opts to be in the center and presents a dilemma or their work with a person or a family and the other genetic counselors listen and respond in ways that new possibilities can emerge for the GC in the center and less directly for the whole group. These sessions have been a chance to share ideas around ways of working and have been both enjoyable and energizing. A fuller account of our collective experiences of narrative counseling supervision will be the subject of a future paper.

## 10 CONCLUSION

As families are at the center of genetic counseling, both the systemic and the narrative working framework can help genetic counselors in their practice.

In this article, we have drawn attention to some of the ways these models work well in the context of genetic counseling. This way of working can be taught to students in Masters genetic counseling training programs, short courses for registered genetic counselors, and also through counseling supervision by qualified Systemic and Narrative practitioners (Table 2).

**TABLE 2:** Summary table

Families are at the center of genetic counseling. Systemic frameworks can help guide our understanding of the dynamic and complex relationships that exist within families and how best to utilize these within genetic counseling. Externalizing conversations creates space for people to consider how they choose to respond in relation to the ‘problem’. Narrative questions can help people to re‐discover existing strengths, values, and hopes that can help to offset the effects of difficulties in their lives. A narrative position can be adopted in any conversation, including medical conversations. We suggest that a narrative position is ideally compatible with the aims of genetic counseling. Multi‐disciplinary team working with family therapists can be successfully incorporated in to routine genetic counseling practice. Narrative practice postulates that people have better lives when they are connected to others, when in relationships with others and able to live their lives according to what is important to them.

## AUTHOR CONTRIBUTIONS

Rhona MacLeod was an invited speaker at the 2nd World Congress of Genetic Counselling and made a substantial contribution to the conception of the paper, the drafting of the work, the editing process, and final approval of the version to be published. Alison Metcalfe made a substantial contribution to the drafting of the work, the editing process, and final approval of the version to be published. Mariangels Ferrer Duch made a substantial contribution to the drafting of the work, the editing process, and final approval of the version to be published.

Agreement is given to be accountable for all aspects of the work in ensuring that questions related to the accuracy or integrity of any part of the work are appropriately investigated and resolved.

## COMPLIANCE WITH ETHICAL STANDARDS

Disclosures Some details have been changed to protect the participants’ identity in the case study.

### Conflict of interest

Rhona MacLeod, Alison Metcalfe, and Mariangels Ferrer Duch declare that they have no conflict of interest.

### Human studies and informed consent

All procedures followed were in accordance with the ethical standards of the responsible committee on human experimentation (international and national) and with the Helsinki Declaration. Informed consent was obtained from all patients for being included in the study conducted by Spiers et al., 2020.

### Animal studies

No non‐human animal studies were carried out by the authors for this article.

### Data sharing and data accessibility

The datasets generated for this study are available on request to the corresponding author.
